# Effects of temperature on mortality in Chiang Mai city, Thailand: a time series study

**DOI:** 10.1186/1476-069X-11-36

**Published:** 2012-07-09

**Authors:** Yuming Guo, Kornwipa Punnasiri, Shilu Tong

**Affiliations:** 1 School of Public Health, Queensland University of Technology, Kelvin Grove, Brisbane, Queensland, Australia; 2 Department of Heath, Ministry of Public Heath, Nonthaburi, Thailand

**Keywords:** Mortality, Cardiovascular, Respiratory, Temperature, Time series analysis

## Abstract

**Background:**

The association between temperature and mortality has been examined mainly in North America and Europe. However, less evidence is available in developing countries, especially in Thailand. In this study, we examined the relationship between temperature and mortality in Chiang Mai city, Thailand, during 1999–2008.

**Method:**

A time series model was used to examine the effects of temperature on cause-specific mortality (non-external, cardiopulmonary, cardiovascular, and respiratory) and age-specific non-external mortality (<=64, 65–74, 75–84, and > =85 years), while controlling for relative humidity, air pollution, day of the week, season and long-term trend. We used a distributed lag non-linear model to examine the delayed effects of temperature on mortality up to 21 days.

**Results:**

We found non-linear effects of temperature on all mortality types and age groups. Both hot and cold temperatures resulted in immediate increase in all mortality types and age groups. Generally, the hot effects on all mortality types and age groups were short-term, while the cold effects lasted longer. The relative risk of non-external mortality associated with cold temperature (19.35°C, 1^st^ percentile of temperature) relative to 24.7°C (25^th^ percentile of temperature) was 1.29 (95% confidence interval (CI): 1.16, 1.44) for lags 0–21. The relative risk of non-external mortality associated with high temperature (31.7°C, 99^th^ percentile of temperature) relative to 28°C (75^th^ percentile of temperature) was 1.11 (95% CI: 1.00, 1.24) for lags 0–21.

**Conclusion:**

This study indicates that exposure to both hot and cold temperatures were related to increased mortality. Both cold and hot effects occurred immediately but cold effects lasted longer than hot effects. This study provides useful data for policy makers to better prepare local responses to manage the impact of hot and cold temperatures on population health.

## Background

Many studies have demonstrated that both hot and cold temperatures had adverse effects on mortality. For example, elevated ambient temperature and heat-waves were associated with excess deaths in 86 US cities [1]. High temperatures had significant impacts on deaths from all causes, chronic bronchitis, pneumonia, ischemic heart disease, and cerebrovascular disease in England and Wales [2]. McMichael et al. found a U-shaped temperature-mortality relationship in developing countries, with strong evidence of increased deaths on hot and cold days [3]. Chung et al. found an increase in mortality associated with elevated average temperature in Seoul, Beijing, Tokyo and Taipei in Asia [4].

Current knowledge of the health effects of temperature on mortality is mainly from developed countries [5,6], particularly from regions with temperate climates. Few studies were conducted in developing countries, particularly in tropical regions [3]. Developing countries are more sensitive to climate change, as they have poor public health infrastructure and more vulnerable populations [3].

Thailand is one of the countries experiencing a significant increase of temperature over recent years. According to the report of Meteorological Department of Thailand [7], ambient temperature significantly increased from 1975 to 2005 by approximately 1°C. Model projections show that the temperature will continue to increase in this century. Meanwhile, respiratory and cardiovascular diseases have become a serious public health issue in Thailand. They are among the top five leading causes of death and disability in Thailand [8]. In particular, Chiang Mai city has the highest mortality and morbidity rates for respiratory disease in Thailand. The incidence and mortality of cardiovascular disease have increased slightly every year [9].

Few studies have examined the impacts of temperatures on cardiovascular, respiratory, and age-specific mortality in Chiang Mai city, Thailand. It is necessary to assess the relationship between temperature and cardiovascular and respiratory diseases in order to better manage this increasing health problem. In this study, we examined the effects of temperature on different types of mortality (including non-external, cardiopulmonary, cardiovascular, and respiratory) and age-specific non-external mortality in Chiang Mai city during 1999–2008.

## Materials and method

### Data

Chiang Mai city is located in the north of Thailand (latitude 18°47'N and longitude 098°59'E). There were 1,603,220 population with 790,107 (49.28%) men and 813.113 (50.72%) women in 2008 [10]. The major businesses in Chiang Mai city are trade, services, and agriculture. The topography of Chiang Mai city is mountain rimmed basin with poor ventilation. Chiang Mai city has grown rapidly in the past decade. It is the area with severe air pollution. Generally, the major ambient pollutants are particulate matter and ozone [11].

We collected daily mortality data from Chiang Mai city during 1999 to 2008. The mortality data were extracted from a computerized file of death certificates provided by the Bureau of Policy and Strategy, Ministry of Public Health, Thailand. These cases were residents in Chiang Mai city. The causes of death were coded according to the 10^th^ Revision of the International Classification of Disease (ICD10). The mortality data were stratified into four cause-specific categories: non-external mortality (A00–R99), cardiopulmonary mortality (I00–I99 and J00–J99), cardiovascular mortality (I00–I99), and respiratory mortality (J00–J99). For cardiopulmonary and respiratory mortality, influenza (J10–J11) was excluded, as it was a potential confounder in the data analyses. We also stratified non-external mortality into four age groups (<=64, 65–74, 75–84, and > =85 years).

Daily data on minimum temperature (minimum value between 00:00 and 9:00 am) and maximum temperature (maximum value between 12:00 and 9: 00 pm) (degrees Celsius) and relative humidity were obtained from the Department of Meteorology at Chiang Mai station. Daily data on average particular matter ≤ 10 μm (PM_10_) (μg/m^3^) and daily average ozone (O_3_) (ppb) were collected from Yuparat School monitoring station and Provincial Hall monitoring station. The daily averaged concentrations of air pollution from the two stations were used in our analysis.

### Statistical models

In this study, we used a Poisson regression model combined with a distributed lag non-linear model (DLNM) to examine the impact of temperature on mortality [12,13]. We used a quasi-Poisson function that allows for over-dispersion in daily deaths. We controlled for PM_10_ (moving average of lags 0–7), O_3_ (moving average of lags 0–7) and relative humidity (moving average of lags 0–7) using a natural cubic spline with 3 degrees of freedom (*df*), as these variables are potential confounders of the association between temperature and mortality [14-16]. We controlled for day of the week as a category variable. We controlled for season and long-term trend using a natural cubic spline with 7 *df* per year for time, as the estimated effects of temperature were then stabilized.

Many studies have shown that the extreme temperature can not only affect current day’s mortality but also influence the several following days’ mortality (lag effect) [17-19]. Also, the relationship between temperature and mortality is non-linear [19]. Therefore, we used a DLNM to examine the non-linear and delayed effects of temperature on cause-specific and age-specific mortality. The DLNM is developed on the basis of “cross-basis” function, which allows simultaneously estimating the non-linear effect of temperature at each lag and the non-linear effects across lags. The DLNM can show the relationship between temperature and mortality at each temperature point and lag. The DLNM can calculate cumulative effect in the existence of delayed contributions. [20].

We used a “natural cubic spline-natural cubic spline” DLNM to examine the non-linear temperature effect using a 5 *df* natural cubic spline and the lagged effect using a 4 *df* natural cubic spline. A maximum lag of 21 days was used to model the effect of temperature on mortality, because previous studies suggest that the effects of cold temperature last about ten days and the effects of high temperature are usually acute and short term [17,19,21]. We placed spline knots at equal spaces in the temperature range, and placed spline knots at equal intervals in the log scale of lags using the default setting of DLNM. The median value of temperature was used as the reference value to calculate the relative risks.

To examine the hot effect on cause-specific and age-specific mortality, we evaluated the relative risk of cause-specific and age-specific mortality associated with high temperature (31.7°C, 99^th^ percentile of temperature) relative to the 75^th^ percentile of temperature (28°C). To examine the cold effect on cause-specific and age-specific mortality, we evaluated the relative risk of cause-specific and age-specific mortality associated with cold temperature (19.35°C, 1^st^ percentile of temperature) relative to the 25^th^ percentile of temperature (24.7°C).

We evaluated the model fit using Akaike's Information Criterion for quasi-Poisson (Q-AIC). Our initial results showed that mean temperature (average of maximum and minimum temperatures) was a better predictor than maximum and minimum temperatures. In addition, mean temperature represents the exposure throughout whole day and night and can be straightforwardly interpreted for decision making purpose [22]. Therefore, mean temperature was used in this study.

To check the main findings of this study, sensitivity analyses were performed by changing *df* (6–15 per year) for time to control for season. We changed *df* (4–7) for humidity, PM_10_, and O_3_, and varied the maximum lags from 22 to 30 days for the DLNM.

All the analyses were performed using R software (version 2.12.1). The “dlnm” package was used to perform DLNM [23].

## Results

During the study period, there were 129,254 non-external deaths, 11,746 (9.09%) from cardiovascular disease and 10,839 (8.39%) from respiratory disease. Table 1 shows the descriptive statistics for mortality, air pollution and weather conditions. There was an average of 35, 6, 3 and 3 cases per day for non-external, cardiopulmonary, cardiovascular and respiratory mortality, respectively. The average daily temperature and relative humidity were 26.24°C (range 13.3–33.5°C) and 70.85 (range 41–95), respectively. The mean concentration of daily PM_10_ and O_3_ was 52.89 μg/m^3^ (range 12.65–356.70) and 17.39 ppb (range 12.65–356.70), respectively.

**Table 1:** Summary statistics for mean temperature, relative humidity, air pollutants, and mortality in Chiang Mai, Thailand, during 1999–2008

Variables	Mean	SD	Min	Percentile			Max
				25	50	75	
Temperature (°C)	26.2	2.7	13.3	24.7	26.8	28.0	33.5
O_3_ (ppb)	17.4	8.2	1.5	11.5	15.5	22.0	56.0
PM_10_ (μg/m^3^)	52.9	35.5	12.7	29.7	42.0	63.3	356.7
Relative humidity (%)	70.8	8.8	41.0	65.0	71.0	77.0	95.0
Non-external mortality	35	7.36	3	31	35	40	65
Cardiopulmonary mortality	6	2.7	0	4	6	8	17
Cardiovascular mortality	3	1.9	0	2	3	4	12
Respiratory mortality	3	1.8	0	2	3	4	11
Non-external mortality < =64 years	17	5.4	1	13	17	21	39
65–74 years	7	2.9	0	5	7	9	19
75–84 years	7	3.0	0	5	7	9	20
> = 85 years	3	1.9	0	2	3	5	11

**Figure 1:**
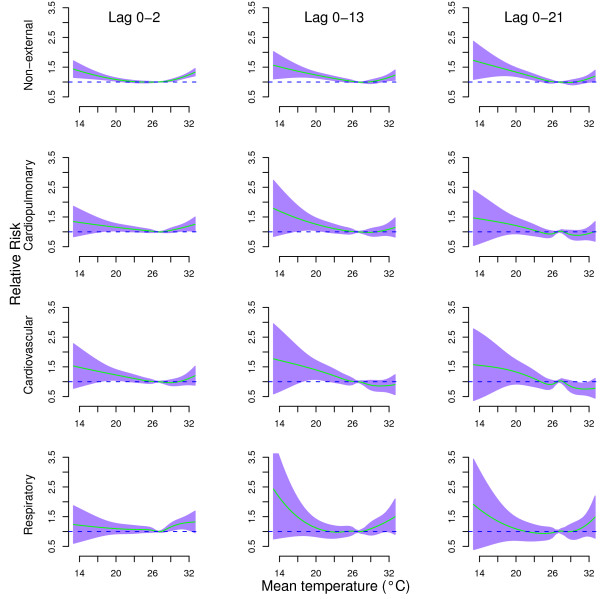
**Relative risks of cause-specific mortality by mean temperature (°C) at lag 0–2 (left), lag 0–13 (middle), and lag 0–21 (right), using a “natural cubic spline-natural cubic spline” DLNM with 5 degrees of freedom natural cubic spline for temperature and 4 degrees of freedom for lag.** The reference value was median temperature (26.8°C). The air pollution, relative humidity, day of the week, and season were controlled for

Figure 1 shows the cumulative effects of temperature on cause-specific mortality at lags 0–2, lags 0–13, and lags 0–21. For lags 0–2, both extreme cold and hot temperatures increased the risks of all mortality types. The cumulative effects of extreme cold temperature on cause-specific mortality at lags 0–13, and lags 0–21 were higher than lags 0–2, while the cumulative effects of extreme high temperature on cause-specific mortality at lags 0–13, and lags 0–21 were lower than lags 0–2, except for respiratory mortality. Generally, the temperature effects on cause-specific mortality were similar at lags 0–13 and lags 0–21, which suggests the temperature effect on cause-specific mortality was stable after the lag of 13 days. For lags 0–21, the relationships between temperature and all mortality types were non-linear.

**Figure 2:**
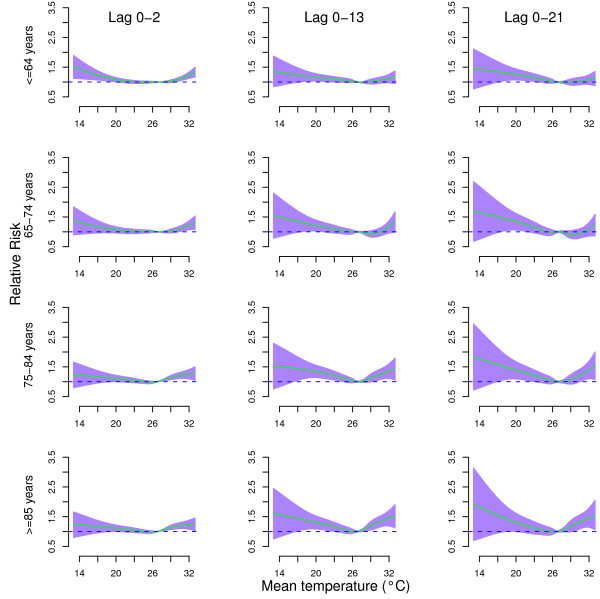
**Relative risks of age-specific non-external mortality by mean temperature (°C) at lag 0–2 (left), lag 0–13 (middle), and lag 0–21 (right), using a “natural cubic spline-natural cubic spline” DLNM with 5 degrees of freedom natural cubic spline for temperature and 4 degrees of freedom for lag.** The reference value was median temperature (26.8°C). The air pollution, relative humidity, day of the week, and season were controlled for

Figure 2 shows the cumulative effects of temperature on age-specific mortality at lags 0–2, lags 0–13, and lags 0–21. The effects of temperature on all age groups were non-linear. The temperature effects on age-specific mortality were similar at lags 0–13 and lags 0–21.

**Figure 3:**
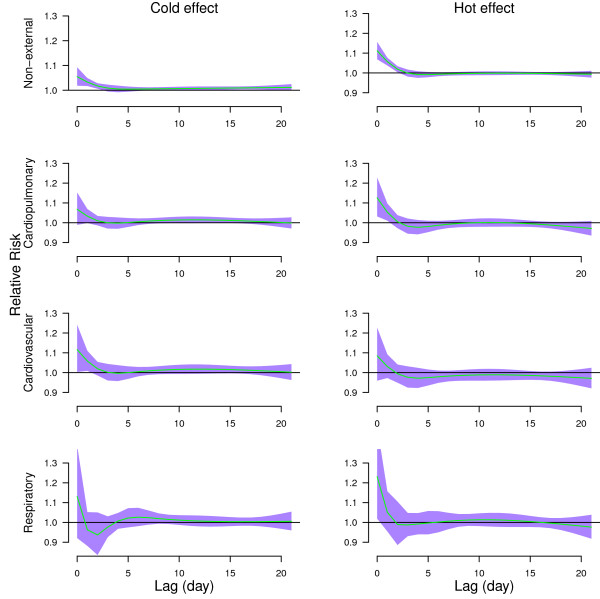
**The estimated cold and hot effects of mean temperature on cause-specific mortality along the lag days, using 4 degrees of freedom natural cubic spline for lag.** The cold effect (left) was estimated by 1^st^ percentile of temperature (19.35°C) relative to 25^th^ percentile of temperature (24.7°C)). The hot effect (right) was estimated by 99^th^ percentile of temperature (31.7°C) relative to 75^th^ percentile of temperature (28°C). The green lines are mean relative risks, and purple regions are 95% confidence intervals

Figure 3 shows the effects of high temperature (31.7°C, 99^th^ percentile) relative to the 75^th^ percentile of temperature (28°C), and the effects of cold temperature (19.35°C, 1^st^ percentile) relative to the 25^th^ percentile of temperature (24.7°C) on non-external, cardiopulmonary, cardiovascular, and respiratory mortality along the lags. Both hot and cold effects occurred immediately for all mortality types. Cold effects lasted longer than hot effects except for respiratory mortality. Hot effects-related excesses of non-external and cardiopulmonary mortality were followed by deficits in mortality (mortality displacement).

**Figure 4:**
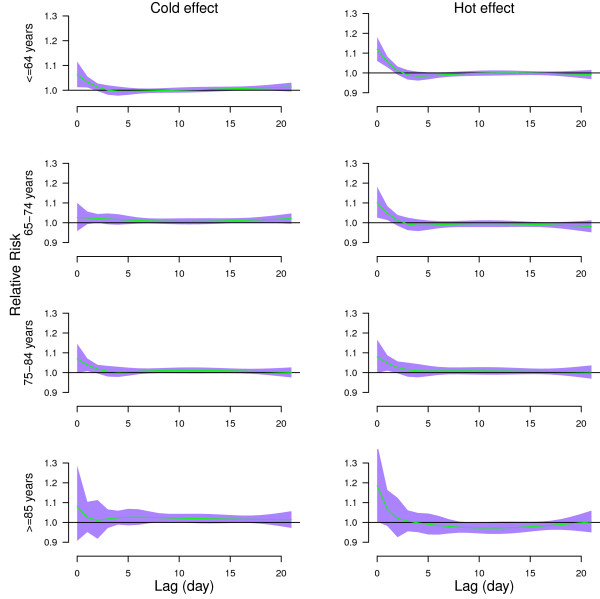
**The estimated cold and hot effects of mean temperature on age-specific non-external mortality along the lag days, using 4 degrees of freedom natural cubic spline for lag.** The cold effect (left) was estimated by 1^st^ percentile of temperature (19.35°C) relative to 25^th^ percentile of temperature (24.7°C)). The hot effect (right) was estimated by 99^th^ percentile of temperature (31.7°C) relative to 75^th^ percentile of temperature (28°C). The green lines are mean relative risks, and purple regions are 95% confidence intervals

Figure 4 shows the effects of high temperature (31.7°C, 99^th^ percentile) relative to the 75^th^ percentile of temperature (28°C), and the effects of cold temperature (19.35°C, 1^st^ percentile) relative to the 25^th^ percentile of temperature (24.7°C) on age-specific mortality along the lags. Both hot and cold effects were acute for all age groups. Acute hot effects were followed by mortality displacement for people aged > = 85 years.

We calculated the cumulative effects of cold temperature on cause-specific mortality and age-specific non-external mortality along the lags, with 1^st^ percentile of temperature (19.35°C) relative to 25^th^ percentile of temperature (24.7°C) (Table 2). The cold temperature was significantly associated with the risk of all mortality types and age groups, except for respiratory mortality.

**Table 2:** **The cumulative effects of cold temperature on cause-specific mortality and age-specific non-external mortality, with 1**^**st**^**percentile of temperature (19.35°C) relative to 25**^**th**^**percentile of temperature (24.7°C)**

Lag	Relative risk (95% CI)			
	Non-external	Cardiopulmonary	Cardiovascular	Respiratory
Lag 0	1.05(1.02,1.09)	1.07(0.99,1.15)	1.12(1.00,10.24)	1.13(0.92,1.39)
Lag 0-1	1.09(1.04,1.15)	1.10(0.99,1.23)	1.18(1.02,1.37)	1.09(0.91,1.31)
Lag 0-2	1.11(1.05,1.17)	1.12(1.00,1.25)	1.20(1.03,1.41)	1.02(0.86,1.21)
Lag 0-3	1.12(1.06,1.18)	1.11(1.00,1.24)	1.21(1.03,1.41)	1.00(0.84,1.19)
Lag 0-7	1.14(1.08,1.21)	1.13(0.99,1.28)	1.22(1.02,1.46)	1.08(0.88,1.32)
Lag 0-13	1.19(1.10,1.29)	1.22(1.04,1.45)	1.34(1.05,1.69)	1.15(0.89,1.49)
Lag 0-21	1.29(1.16,1.44)	1.29(1.02,1.63)	1.45(1.04,2.02)	1.20(0.84,1.71)
	<=64 years	65–74 years	75–84 years	> = 85 years
Lag 0	1.06(1.01,1.12)	1.03(0.96,1.10)	1.07(1.00,1.15)	1.08(0.91,1.29)
Lag 0-1	1.10(1.03,1.18)	1.05(0.95,1.16)	1.11(1.01,1.22)	1.11(0.95,1.30)
Lag 0-2	1.11(1.04,1.19)	1.07(0.97,1.19)	1.13(1.02,1.25)	1.12(0.97,1.29)
Lag 0-3	1.11(1.04,1.19)	1.10(0.99,1.21)	1.14(1.03,1.26)	1.14(0.98,1.32)
Lag 0-7	1.10(1.01,1.19)	1.15(1.03,1.29)	1.16(1.04,1.31)	1.26(1.06,1.50)
Lag 0-13	1.11(0.99,1.23)	1.21(1.04,1.40)	1.24(1.06,1.45)	1.43(1.14,1.79)
Lag 0-21	1.19(1.02,1.38)	1.35(1.10,1.67)	1.30(1.05,1.61)	1.62(1.18,2.22)

We calculated the cumulative effects of high temperature on cause-specific mortality and age-specific non-external mortality along the lags, with 99^th^percentile of temperature (31.7°C) relative to 75^th^ percentile of temperature (28°C) (Table 3). For the short lags (e.g., lag 0 to lag 0–3), the high temperature was significantly associated with the risk of all mortality types and age groups, except for cardiovascular mortality. When we estimated the overall effect of lags 0–21, the relative risks were not statistically significant for all mortality types except for non-external mortality in all age groups and those among 75–84 years.

**Table 3:** **The cumulative effects of high temperature on cause-specific mortality and age-specific non-external mortality, with 99**^**th**^**percentile of temperature (31.7°C) relative to 75**^**th**^**percentile of temperature (28°C)**

Lag	Relative risk (95% CI)			
	Non-external	Cardiopulmonary	Cardiovascular	Respiratory
Lag 0	1.11(1.07,1.16)	1.13(1.03,1.23)	1.08(0.96,1.23)	1.23(1.02,1.49)
Lag 0-1	1.18(1.11,1.24)	1.19(1.05,1.34)	1.12(0.94,1.33)	1.29(1.07,1.57)
Lag 0-2	1.20(1.13,1.27)	1.19(1.04,1.36)	1.11(0.92,1.34)	1.28(1.05,1.57)
Lag 0-3	1.20(1.12,1.27)	1.17(1.02,1.34)	1.09(0.90,1.31)	1.26(1.02,1.57)
Lag 0-7	1.16(1.08,1.25)	1.10(0.93,1.30)	0.99(0.78,1.25)	1.26(0.97,1.64)
Lag 0-13	1.15(1.05,1.26)	1.10(0.90,1.34)	0.92(0.69,1.21)	1.36(1.00,1.83)
Lag 0-21	1.11(1.00,1.24)	0.98(0.77,1.23)	0.78(0.56,1.09)	1.28(0.90,1.83)
	<=64 years	65–74 years	75–84 years	> = 85 years
Lag 0	1.12(1.06,1.18)	1.11(1.03,1.20)	1.08(1.00,1.17)	1.18(1.00,1.40)
Lag 0-1	1.18(1.09,1.27)	1.17(1.05,1.31)	1.13(1.01,1.26)	1.27(1.07,1.50)
Lag 0-2	1.19(1.10,1.30)	1.19(1.06,1.34)	1.16(1.03,1.31)	1.29(1.08,1.54)
Lag 0-3	1.18(1.09,1.29)	1.19(1.06,1.35)	1.18(1.04,1.33)	1.30(1.08,1.57)
Lag 0-7	1.12(1.01,1.25)	1.17(1.01,1.36)	1.22(1.05,1.42)	1.24(0.99,1.56)
Lag 0-13	1.11(0.98,1.26)	1.15(0.96,1.38)	1.28(1.07,1.53)	1.06(0.81,1.38)
Lag 0-21	1.08(0.93,1.25)	1.08(0.87,1.33)	1.33(1.08,1.64)	0.96(0.70,1.32)

### Sensitivity analysis

We changed *df* (6–15 per year) for time to control for season, which gave similar results (data not shown). We changed *df* (3–7) for humidity, PM_10_, and O_3_, and the estimated effects of temperature were not substantially changed. In addition, we changed the maximum lag from 22 to 30 days, which gave similar results (data not shown). The models used in this study seemed to have adequately captured the main effects of temperature on mortality.

## Discussion

In this study, we examined the effects of temperature on cause-specific and age-specific mortality in Chiang Mai city, Thailand, during 1999–2008. We found that the temperature-mortality relationships were non-linear for all mortality types and age groups. Both extreme cold and hot temperatures had negative impacts on mortality. Both cold and hot effects occurred acutely for all mortality types. Cold effects lasted longer than hot effects.

In general, the relationship between temperature and cause-specific mortality is consistent with previous studies worldwide [3,24]. This study has also explored the lag effects of temperature on case-specific mortality using DLNM up to 21 days. We found that both hot and cold temperatures were associated with increased non-external deaths. The strongest temperature-mortality relationship was at lag 0. Lag structures in the relationship between temperature and mortality have been reported in several studies [17,25-27]. Previous studies also reported a similar lag effect of temperature on mortality [17,19,21,28]. Many studies showed that hot temperatures had short term effects on mortality and morbidity [17,25-27], while the cold effect were delayed and lasted for several days [6,29]. The acute cold effects in this study might be due to the tropic climatic pattern in Chiang Mai city as people are not accustomed to cold weather.

Even though this study found that both high and low temperatures resulted in immediate increases in mortality, the effects of hot and cold temperatures were different. There were acute and short-term effects of hot temperature on mortality, while the effects of cold temperature persisted for several days. In addition, we found hot effects had mortality displacement for non-external and cardiopulmonary mortality, and people aged > = 85 years. This finding is consistent with several previous studies [17,19,30-32]. Hence, it suggests that even in a tropic climate, hot temperature has short term acute effect and cold temperature has relatively long, lagged effects on mortality. Studies in Europe and USA have confirmed that cold temperatures associated with risk in mortality, and that cold effects on mortality in warmer cities were higher than cold cities [33]. People living in rooms without heating, and those wearing fewer clothers had higher cold related mortality [34].

Many studies have examined the relationship between temperature and mortality in Australia, Europe, and USA [35-39], but few are from Thailand [3,40]. Studies showed that effects of temperature on mortality varied by population and region [37,41-44]. The relationship between temperature and non-external mortality in this study was similar as a previous study conducted in Chiang Mai city [3]. In Chiang Mai city, both extreme cold and high temperatures had significant effects on non-external mortality. Studies showed that cities with median or lower income (e.g., Bangkok, Mexico City, São Paulo, Delhi, Santiago, and Cape Town) had significant hot and cold effects on non-external mortality [19].

The findings in this study were biologically plausible. The human body regulates the heat exchange between the body and ambient temperature by physiological control. When the body is exposed to extreme high temperatures, the thermoregulation system may fail. The reason may be related to dehydration, salt depletion and increased surface blood circulation [45]. High temperature is also related to elevated blood viscosity, cholesterol levels and sweating thresholds, which may also be the cause of heat-related mortality [46]. Extreme cold temperatures are related to the increase in the heart rate, peripheral vasoconstriction, blood pressure, blood cholesterol levels, plasma fibrinogen concentrations, and platelet viscosity [47,48].

This study has two major strengths. Firstly, this study investigated the effects of temperature on non-external, cardiopulmonary, cardiovascular and respiratory mortality, and age-specific non-external mortality using an advanced statistical approach (DLNM) in Chiang Mai city, Thailand. The advanced statistical approach can flexibly examine effects of temperature on mortality. For example, it can smooth temperature and lag at the same time. Secondly, we used ten years’ data which had high quality (no missing data for mortality). We also adjusted for a range of confounders including relative humidity and air pollutants. However, this study also has some limitations. This is an ecological study in which exposure misclassification might occur as detailed spatial information was unavailable in this study. We only used data from Chiang Mai city to examine the effects of temperature on mortality, so the findings may be difficult to generalize to other areas. We did not control for barometric pressure, wind direction, and wind speed, as these data were not available.

## Conclusions

This study examined the effects of temperature on cause-specific and age-specific mortality in Chiang Mai city, Thailand. The relationships between temperature and cause-specific and age-specific mortality were non-linear. Both cold and hot temperatures were associated with increased risk in mortality. Both cold and hot effects occurred immediately. Cold effects lasted longer than hot effects. Cold related mortality risk in oldest people (aged > =85 years) was higher than other age groups. These results demonstrate that temperature is an important environmental hazard in Chiang Mai city.

## Abbreviations

AIC: Akaike's Information Criterion; CI: Confidence interval; Df: Degree of freedom; DLNM: Distributed lag non-linear model; ICD10: International classification of cisease, tenth revision; PM10: Particulate matter ≤10 μm; RR: Relative risk; SD: Standard deviation; SO2: Sulfur dioxide.

## Competing interests

The authors declare that they have no competing interests.

## Authors' contributions

GY and PK conducted the study, performed data analysis and drafted the manuscript; TS conceived the study and contributed to the study design, reviewed and edited the manuscript. All authors read and approved the final manuscript.
